# Investigations of injection strategies to use heparinized normal saline instead of contrast media for intracoronary optical coherence tomography imaging

**DOI:** 10.1177/02676591241264116

**Published:** 2024-06-21

**Authors:** Aiste Zebrauskaite, Eduard Tsybulskyi, Ignas Simanauskas, Gabriele Zebrauskaite, Greta Ziubryte, Rasa Ordiene, Ramunas Unikas, Gediminas Jarusevicius, Scott Andrew Harding

**Affiliations:** 1 Department of Cardiology, 230647 Hospital of Lithuanian University of Health Sciences Kaunas Clinics, Kaunas, Lithuania; 2 Faculty of Medicine, 230647 Lithuanian University of Health Sciences, Kaunas, Lithuania; 3 Department of Cardiology, 230647 Kaunas Hospital of Lithuanian University of Health Sciences, Kaunas, Lithuania; 4 Institute of Cardiology, Kaunas, Lithuania; 5 Department of Cardiology, Wellington Hospital, Wellington, New Zealand

**Keywords:** optical coherence tomography, intravascular imaging, saline OCT, contrast usage lowering strategies, complex PCI

## Abstract

**Background:**

The benefits of intravascular imaging-guided percutaneous coronary interventions (PCI) are well established. Intravascular imaging guidance improves short- and long-term outcomes, especially in complex PCI. Optical coherence tomography (OCT) has a higher resolution than intravascular ultrasound. However, the usage of OCT is mainly limited by the need to use contrast for flushing injections, which increases the risk of contrast-induced acute kidney injury, especially in patients with underlying chronic kidney disease. The aim of this study was to prove that flushing techniques with normal saline instead of contrast can be used in OCT imaging and can generate high-quality images.

**Methods:**

This prospective single-center observational study included patients with indications for OCT-guided PCI. For OCT pullbacks, heparinized saline was injected by an automatic pump injector at different rates, and additional extension catheters for selective coronary artery engagement were used at the operator’s discretion. Recordings were made using the Ilumien Optis OCT system (Abbott) and the Dragonfly (Abbott) catheter and were analyzed at 1-mm intervals by two operators. Pullbacks were categorized as having optimal, acceptable, or unacceptable imaging quality. A clinically usable run was determined if >75% of the region of interest length was described as having optimal or acceptable imaging quality.

**Results:**

A total of 32 patients were enrolled in the study; 47 different lesions were assessed before and after PCI. In total, 91.5% of runs were described as clinically suitable for use.

**Conclusion:**

Heparinized saline injections for OCT imaging are effective in generating good-quality OCT images suitable for clinical use.

## Introduction

Approximately 7% of patients undergoing percutaneous coronary intervention (PCI) experience contrast-induced nephropathy (CIN).^1^ However, in patients with advanced kidney disease, the incidence of CIN increases to around 30%.^1,2^ Contrast-induced nephropathy has been strongly associated with adverse clinical outcomes, including death, the need for hemodialysis, increased hospital costs, and lengths of stay.^1–6^ The ratio of the contrast media (CM) volume used to creatinine clearance is one of the strongest predictors of CIN development following PCI.^7,8^ Therefore, to reduce the incidence of CIN, it is important to focus on strategies to reduce the volume of CM used, particularly in patients with chronic kidney disease (CKD).

Multiple studies and meta-analyses have shown that compared to coronary angiography alone, the use of intravascular imaging techniques for PCI guidance reduces the risk of cardiovascular death and adverse events.^9–17^ Optical coherence tomography (OCT) has superior resolution compared to intravascular ultrasound (IVUS).^18,19^ Optical coherence tomography techniques have been shown to more accurately assess vessel morphology, sizing, and complications following stenting compared to angiography or IVUS.^19,20^ This makes OCT a powerful tool for optimizing the outcomes of percutaneous coronary intervention, particularly in complex lesions.^17,19^

Optical coherence tomography is a high-resolution (10 μm) catheter-based intracoronary imaging technique. Similar to IVUS, OCT provides cross-sectional images of the vessel. However, instead of sound waves, OCT uses near-infrared light waves for tissue analysis, enabling visualization of coronary lesions with microscopic precision.^21,22^ Furthermore, red blood cells interfere with the propagation of infrared light; therefore, OCT requires displacement of the red blood cells from the vessel lumen during image acquisition to avoid artifacts that may limit good visualization of the coronary artery wall.^23^ This result was previously achieved by relatively complex techniques, such as occluding the coronary lumen with an inflated balloon and simultaneously flushing the vessel with saline through the balloon catheter. Currently, non-occlusive techniques that involve flushing the coronary artery with CM to displace the blood pool during the vessel’s scan allow full vessel visualization without occlusion.^21^ CM is nephrotoxic, and its use during OCT significantly adds to the total volume of CM used during PCI. Depending on the size of the vessel, 11–17 mL of contrast media is used during one OCT pullback.^23,24^ Consequently, the usage of OCT is limited in patients with renal impairment.^25^ Normal saline is an alternative agent that could potentially be used for the clearance of red blood cells during OCT. It is not nephrotoxic, is cheap, and easy to use. However, normal saline is less viscous compared to CM and will require an intracoronary injection of larger volumes at higher rates. No protocol for normal saline injections in OCT imaging has been published to date. The aim of our study was to assess if it is possible to get good-quality images by using normal saline for OCT imaging, to establish recommendations for injection rate, and to evaluate the safety of this procedure.

## Methods

This was a prospective single-center observational study performed in the Clinic of Cardiology at the Hospital of Lithuanian University of Health Sciences Kaunas Clinics between September 2022 and September 2023. The study protocol was reviewed and approved by the regional bioethics committee. The study was conducted in accordance with the Declaration of Helsinki and approved by the Kaunas Regional Biomedical Research Ethics Committee (protocol code BE-2-7, dated February 22, 2022).

Informed consent was obtained from all patients before enrollment in the study. The primary endpoint was to assess the efficacy of saline OCT in generating high-quality images in anatomically selected coronary arteries. The secondary endpoint was to create a protocol for saline injection into the coronary artery depending on the visual angiographic diameters of the vessel and to assess angiographic features that would be useful in identifying anatomical angiographic parameters to predict the suitability of the vessel to obtain good-quality OCT images. We enrolled patients for whom intravascular imaging-guided PCI was indicated. To be enrolled in the study, patients had to be ≥ 18 years old and willing to participate by signing an informed consent form. Patients with acute ST-elevation myocardial infarction were not eligible. Patients with specific angiographic findings that could affect the quality of imaging were not included: an angiographically small vessel <2.0 mm in diameter, an angiographically large or ectatic vessel >5.0 mm in diameter, ostial and left main lesions, very distal lesions, long lesions >45 mm in length, tortuous and severely calcified lesions. During the study period, 3585 PCI procedures were performed in our center. During this period, heparinized saline was used for all OCT imaging injections. All 32 patients were enrolled in the study. Before enrollment, patients had to meet all angiographical study inclusion criteria. For patients who did not meet angiographical study inclusion criteria, intravascular ultrasound was performed instead of OCT.

For the OCT-guided PCI procedure, coronary arteries were planned to be imaged at least twice: before PCI for vessel and atherosclerosis morphological assessment, and after PCI to assess stent expansion, apposition, and stenting-related complications.

Heparinized saline was injected into the coronary system for blood clearance during OCT image acquisition. All OCT pullbacks were performed using an automatic syringe injector (Medrad, Germany). The flushing injections might be performed manually or by using the automatic pump injector, as per the operator’s preference.^25^ Manual injections have been widely used between operators due to the fast and easy flushing, which does not require any additional preparation. However, automatic pump injections require additional preparation and precise de-airing of the system; the parameters of automatic pump injections are more controlled and all changes can be exactly registered and monitored. Because of the need to monitor all injection parameters carefully, all injections were performed using an automatic pump injector. Due to the lower viscosity of saline, it had to be injected at a higher rate and higher volume compared to CM injections. For selective engagement of the coronary artery, additional extension catheters were available for use under the judgment of the operator (guiding extension catheter or “mother in child/5 in 6 F catheters”). Injections to the right coronary artery (RCA) and selective injections to the left anterior descending (LAD) and the left circumflex (LCX) arteries were done at a rate of 4 mL/s, and nonselective injections to the left coronary artery (LCA) at a rate of 5 mL/s. The operator could perform as many runs as necessary. The speed and volume of injection could be increased further at the operator’s discretion if pull-back imaging quality was not sufficient. Due to the poor viscosity of normal saline, when imaging longer lesions, two runs might be taken to assess the proximal and distal lesion/stent segments. Optical coherence tomography pullbacks last <3.0 s, and no CM is used for flushing; multiple repeated runs are considered to be benign for patients.

Recordings were made using the Ilumien Optis intravascular OCT imaging system (Abbott, Santa Clara, California) using a Dragonfly (Abbott, Santa Clara, California) imaging catheter at a speed of 20 mm/s. Heparinized saline was injected into the coronary system for blood clearance during OCT image acquisition. The guiding catheter was properly engaged before image acquisition. Any remaining CM was withdrawn from the guiding catheter before the controlled injection of heparinized saline. Before every OCT run, 100 μg of intracoronary nitroglycerine (NTG) was given to avoid catheter- or saline-induced coronary artery spasm. Electrocardiographic and hemodynamic changes were observed during pullback.

**Figure 1.**
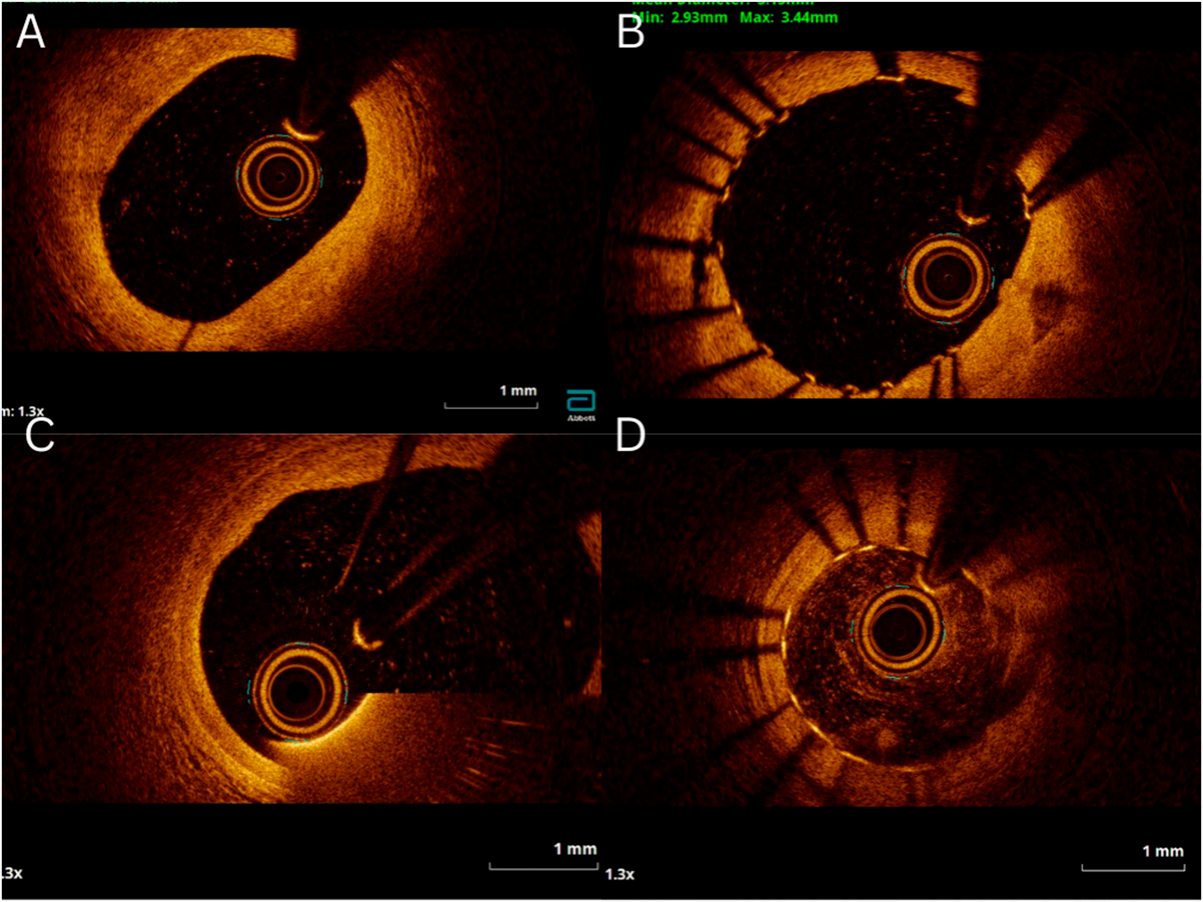
Examples of saline OCT images of different quality. (A and B) optimal imaging quality; (C) acceptable imaging quality; (D) unacceptable imaging quality.

Recordings were analyzed at 1-mm intervals by one or two experienced operators. The assessment of imaging quality was based on imaging clarity, external elastic membrane (EEL) or lumen contour visibility, the system’s ability to perform automatic calculations, and the absence of artifacts or squiring. Optimal imaging (Oi) was defined as a completely clear vessel lumen and visible lumen contour or EEL for 360° of vessel circumference; no blood swirl in the run; and detailed coronary lesion and stent characteristics. Acceptable imaging (Ai) was defined as a visible lumen contour or EEL >270°; visible blood in the run, but not hampering the coronary lesion characteristics and minimum lumen area; a visible proximal and distal reference landing zone; visualization of stent strut apposition, stent edge dissection, and plaque prolapse. Unacceptable imaging (Ui) was defined as a visible lumen contour and/or EEL <270°; no diagnostic information could be obtained, largely because of poor clearance of blood. If one OCT run was not enough to get good-quality imaging along the entire length of the lesion, then the lesion could be divided into halves or thirds, and multiple runs could be performed to assess each region. A clear imaging field (CIF) was defined as a visible lumen contour >270°.^24^ Adequately imaged vessel length was determined as the segment of recording containing all frames with a CIF and further categorized as continuous length or proportional length. A clinically usable run (CUR) was considered suitable for clinical use if >75% of the region of interest length was described as imaging of Oi or Ai quality. Figure 1 shows examples of saline OCT imaging from this study.

Periprocedural complications related to the usage of OCT imaging catheters and/or additional extension catheters were recorded. These included dynamic ECG changes (transient ST depression and transient ST elevation), transient chest pain, transient arrhythmias (bradycardia, ventricular tachycardia, and ventricular fibrillation), iatrogenic coronary artery dissection, no reflow after OCT imaging pullback, and coronary artery spasm. All mentioned complications were registered based on patients’ symptoms, ECG changes, and angiographic views.

Statistical analysis was carried out using SPSS version 24.0. Categorical variables were expressed as percentages, and continuous variables with a normal distribution were expressed as mean ± standard deviation. Continuous variables that did not meet normal distribution criteria were expressed as mean (median). The Shapiro-Wilk and Kolmogorov-Smirnov tests were used to assess data normality. The chi-square test was used to compare categorical variables. Numerical data were assessed using Pearson’s, χ2, and Fisher exact tests. To assess normally distributed data between two groups, the independent-sample t-test was employed; to assess non-normally distributed data, the Mann-Whitney test was used. For comparisons among more than two groups, one-way analysis of variance (ANOVA) and Kruskal-Wallis tests were used accordingly.

## Results

A total of 32 patients, with a mean age of 70.38 ± 8.78 years, were enrolled in the study, of whom 62.5% were male, and 25.0% had a diagnosis of CKD. Among these patients, 47 different lesions were assessed both before and after PCI. The distribution of these lesions by location was as follows: 18 (38.3%) in the LAD, 14 (29.8%) in the LCX artery, and 15 (31.9%) in the RCA.

The detailed characteristics of patients’ demographics and assessed lesions are provided in Table 1. A total of 117 pullbacks were performed before and after PCI; of these, 94 runs (47 pre-PCI and 47 post-PCI) were deemed suitable for final analysis. Prior to PCI, 58 pullbacks were conducted for lesion assessment, with 10 of them deemed unsuitable for analysis and requiring repetition. Among the repeated pre-PCI imaging runs, saline injection speed was increased by 1 to 2 mL/s in 60.0% of cases (6 runs), extension catheters for selective artery engagement were used in 20.0% (2 runs), and an extension catheter with an increased injection speed was used in 20.0% (2 runs). After PCI, a total of 59 runs were performed, with 12 of them being deemed unsuitable for assessment and thus repeated. Among the repeated post-PCI runs, saline injection speed was increased by 1 to 2 mL/s in 63.60% of cases (7 runs), extension catheters for selective artery engagement were used in 8.3% (1 run), and the area of interest was divided into halves and imaged by performing separate runs for each half in 33.3% (4 runs) of cases.

**Table 1.** Detailed characteristics of patients’ demographics and assessed lesions.

Patients’ characteristics	*N* = 32
Demographics	
Age, mean ± SD, years	70.38 ± 8.78
BMI, mean ± SD, kg/m^2^	27.47 ± 2.95
Sex, *n* (%)	
Male	20 (62.5)
Female	12 (37.5)
Smokers, *n* (%)	9 (28.1)
Comorbidities, *n* (%)	
AH	31 (96.9)
Dyslipidemia	31 (96.9)
DM	10 (31.3)
Previous MI	9 (28.1)
Previous percutaneous coronary intervention	11 (34.4)
Previous CABG	2 (6.3)
Baseline renal function	
Baseline serum creatinine, mean ± SD, μmol/L	80.68 ± 19.73
Normal (CrCl ≥60 mL/min), *n* (%)	24 (75.0)
Mild CKD (CrCl 46–59 mL/min), *n* (%)	4 (12.5)
Moderate CKD (CrCl 30–45 mL/min), *n* (%)	2 (6.3)
Severe CKD (CrCl <30 mL/min), *n* (%)	2 (6.3)
Presentation, *n* (%)	
Stable angina	19 (59.4)
Acute coronary syndrome (UA, NSTEMI)	11 (34.4)
Pre-PCI OCT	
Additional catheters, *n* (%):	
Not used	21 (44.7)
Extension guiding catheter	11 (23.4)
5 in 6 F catheter	15 (31.9)
Parameters changed if prior imaging not suitable for assessment (*n* = 10), what was changed, *n* (%):	
Increased injection volume speed	6 (60.0)
Additional catheters used	2 (20.0)
Increased injection volume speed and additional catheters used	2 (20.0)
Visible 5 mm distally of the lesion, *n* (%):	
No	5 (10.6)
Yes	42 (89.4)
Visible 5 mm proximally to the lesion, *n* (%):	
No	8 (17.0)
Yes	39 (83.0)
OCT post-PCI	
Parameters changes if prior imaging is not suitable for assessment: (*n* = 12), *n* (%):	
Increased injection volume speed	7 (58.3)
Additional catheters used	1 (8.3)
Segment of interest divided into two parts, each part assessed by a different OCT run	4 (33.3)
Ability to assess stent expansion, *n* (%):	
Good	38 (80.9)
Acceptable	9 (19.1)
Visible 5 mm proximally to the lesion, *n* (%):	
No	14 (29.8)
Yes	33 (70.2)
Visible 5 mm distal to the lesion, *n* (%)	
No	9 (19.1)
Yes	38 (80.9)

Ai - acceptable imaging; AH - arterial hypertension; BMI - body mass index; CA - coronary artery; CABG - coronary artery bypass grafting; CKD - chronic kidney disease; CrCl - creatinine clearance; CUR – clinically usable run; DM - diabetes mellitus; LAD - left anterior descending; LCX - left circumflex; MI - myocardial infarction; NSTEMI - non-ST-segment MI; OCT - optical coherence tomography; Oi – optimal imaging; PCI - percutaneous coronary intervention; RCA - right coronary artery; UA - unstable angina.

A total of 94 OCT runs were included in the final analysis, with 47 runs conducted pre-PCI and 47 post-PCI, all analyzed frame by frame. Among the 94 runs, 86 (91.4%) were quantified as CUR. Of these, 91.5% of pre-PCI and 91.5% of post-PCI runs were suitable for analysis. However, in the pre-PCI OCT group, Oi quality was obtained in 75.85% (100%) of the assessed lesion length, whereas in the post-PCI OCT group, Oi quality decreased to 58.11% (70.00%) (*p* = .001). Conversely, the percentage of the assessed segment with Ai quality was higher in the post-PCI group (35.66% (15.00%) versus 17.96% (0%) (*p* < .001)). In both groups, only minor parts of the assessed segments were not suitable for analysis (6.62% (0%) and 6.23% (0%) respectively, (*p* > .05)). The lower rate of Oi quality after PCI may be the result of increasing lumen area and imaging lesion length after PCI. Additional extension or “mother-in-child” catheters were used in the assessment of 27 (58.7%) lesions: in 26 cases, catheters were used prior and after PCI, and in one case, only for post-PCI OCT imaging. All additional catheters were used in the LCA system. Detailed analysis of pre-PCI and post-PCI runs is provided in Table 2.

**Table 2.** Data of detailed analysis on pre-PCI and post-PCI OCT technique and imaging.

Characteristics	OCT pre-PCI	OCT post-PCI	*p*
	*n* = 47	*n* = 47	
Imaging sessions performed in order to obtain images suitable for analysis, *n* (%):			
1	36 (76.6)	35 (74.5)	1.000
2	11 (23.4)	11 (23.4)	
3	0 (0.0)	1 (2.1)	
Injection speed, *n* (%):			0.025
4 mL/s	12 (25.5)	7 (14.9)	0.203
5 mL/s	29 (61.7)	22 (46.8)	0.149
6 mL/s	6 (12.8)	17 (36.2)	0.009
7 mL/s	0 (0.0)	1 (2.1)	0.321
Minimal CA/stent diameter assessed on OCT	3.06 ± 0.45	3.10 ± 0.45	0.002
Maximal CA/stent diameter assessed on OCT	3.34 ± 0.43	3.34 ± 0.44	0.422
Lesion/stent length assessed on OCT, *n*, (median)	21.49 (20.00)	25.28 (24.00)	<0.001
CUR *n*, (%)	43 (91.5)	43 (91.5)	1.000
Percentage of Oi and Ai quality of the assessed segment, *n* (median)	93.91 (100)	93.55 (100)	0.601
Oi, *n* (median)	75.85 (100)	58.11 (70)	0.001
Ai, *n* (median)	17.96 (0)	35.66 (15.00)	<0.001
Ui, *n* (median)	6.62 (0)	6.23 (0)	0.983

Ai - acceptable imaging; CA - coronary artery; CUR – clinically usable run; OCT - optical coherence tomography; Oi – optimal imaging; PCI - percutaneous coronary intervention; Ui – unacceptable imaging.

**Figure 2.**
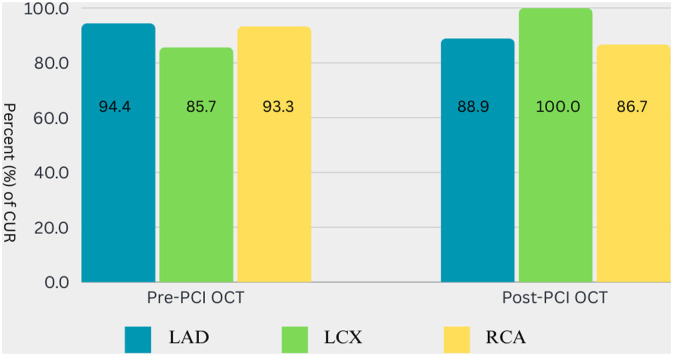
CUR imaging between different coronary arteries. LAD – left anterior descending, LCX – left circumflex, RCA – right coronary artery.

Upon analyzing the OCT assessment across different coronary arteries, there was no statistical significance in the number of runs required to obtain imaging suitable for inclusion in the analysis group. However, in the LCX artery, the percentage of two out of two suitable and included runs was lower compared to the LAD and RCA groups (6 (42.9%) versus 11 (61.1%) and 9 (60.0%), *p* > .05). Extension catheters to improve imaging quality were not needed in RCA arteries. The injection rate of 4 mL/s was used exclusively for selective engagement with extension and mother-in-child catheters; therefore, this rate was never used for imaging the RCA. A higher infusion rate, specifically 6 mL/s, was significantly more often used in the RCA (*p* = .033). While CUR imaging slightly varied across different types of arteries, no statistical significance was observed. Percentage of CUR imaging between different coronary arteries provided in Figure 2. Table 2 displays the percentage of CUR imaging across different coronary arteries, along with injection technique analysis and imaging analysis data in different coronary arteries. Further analysis and comparison of runs across different vessels are provided in Table 3.

**Table 3.** Comparative analysis of LAD, LCX, and RCA imaging technique and imaging analysis data.

Characteristics	LAD	LCX	RCA	*p*
	*n* = 18	*n* = 14	*n* = 15	
Total number of pre- and post-PCI images, *n* (%):				
2	11 (61.1)	6 (42.9)	9 (60.0)	0.767
3	6 (33.3)	7 (50.0)	6 (40.0)	
4	1 (5.6)	1 (7.1)	0 (0.0)	
Additional catheters, *n* (%):				
Not used	3 (16.7)	3 (21.4)	15 (100)	<0.001
Extension guiding catheter	6 (33.3)	5 (35.7)	0 (0.0)	
5 in 6 F catheter	9 (50.0)	6 (42.9)	0 (0.0)	
Parameters changed if prior imaging not suitable for assessment (*n* = 10), what was changed, *n* (%):				
Increased injection volume speed	1 (33.3)	3 (60.0)	2 (100)	1.000
Extension guiding catheter	1 (33.3)	1 (20.0)	0 (0.0)	
Increased injection volume speed and additional catheters used	1 (33.3)	1 (20.0)	0 (0.0)	
Injection speed pre-PCI OCT, *n* (%):				0.035
4 mL/s	7 (38.9)	5 (35.7)	0 (0.0)	0.012
5 mL/s	10 (55.6)	7 (50.0)	12 (80.0)	0.218
6 mL/s	1 (5.6)	2 (14.3)	3 (20.0)	0.480
CUR, *n* (%)	17 (94.4)	12 (85.7)	14 (93.3)	0.672
Visible 5 mm proximally to the lesion, *n* (%):				
No	4 (22.2)	1 (7.1)	3 (20.0)	0.620
Yes	14 (77.8)	13 (92.9)	12 (80.0)	
Visible 5 mm distally to the lesion, *n* (%):				
No	1 (5.6)	4 (28.6)	0 (0.0)	0.030
Yes	17 (94.4)	10 (71.4)	15 (100)	
Number of images performed after PCI, *n* (%):				
1	13 (72.2)	10 (71.4)	12 (80.0)	0.966
2	4 (22.2)	4 (28.6)	3 (20.0)	
3	1 (5.6)	0 (0.0)	0 (0.0)	
Parameters changed if prior imaging not suitable for assessment (*n* = 10), what was changed (*n* = 12), *n* (%):				
Increased injection volume speed	3 (60.0)	2 (50.0)	2 (66.7)	1.000
Additional catheters used	1 (20.0)	0 (0.0)	0 (0.0)	
Segment of interest divided into two parts, each part assessed by a different OCT run	1 (20.0)	2 (50.0)	1 (33.3)	
Visible 5 mm proximal to the lesion, *n* (%):				
No	7 (38.9)	4 (28.6)	3 (20.0)	0.482
Yes	11 (61.1)	10 (71.4)	12 (80.0)	
Visible 5 mm distal to the lesion, *n* (%):				
No	5 (27.8)	3 (21.4)	1 (6.7)	0.335
Yes	13 (72.2)	11 (78.6)	14 (93.3)	
Injection speed post-PCI OCT, *n* (%):				0.085
4 mL/s	4 (22.2)	3 (21.4)	0 (0.00)	0.144
5 mL/s	10 (55.6)	6 (42.9)	6 (40.0)	0.711
6 mL/s	3 (16.7)	5 (35.7)	9 (60.0)	0.033
7 mL/s	1 (5.6)	0 (0.0)	0 (0.0)	1.000
CUR, *n* (%)	16 (88.9)	14 (100)	13 (86.7)	0.534

Ai - acceptable imaging; CUR – clinically usable run; LAD - left anterior descending; LCX - left circumflex; OCT - optical coherence tomography; Oi – optimal imaging; PCI - percutaneous coronary intervention; RCA - right coronary artery; Ui – unacceptable imaging.

**Figure 3.**
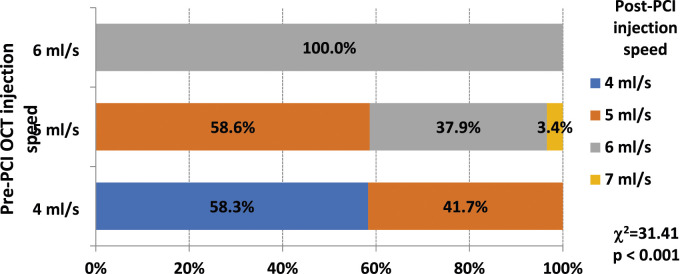
Alterations of post-PCI OCT injections speed increasement in comparison of pre-PCI OCT injection speed. OCT – optical coherence tomography; PCI - percutaneous coronary intervention.

The speed of saline injection to the RCA pre-PCI varied from 5 to 6 mL/s, while to the LCA, it ranged from 4 to 6 mL/s; the speed of 4 mL/s was used as the starting speed for selective injections with extension catheters. We found that post-PCI imaging runs had to be performed with the same or higher injection volume compared to pre-PCI runs, with speeds varying between 4 and 7 mL/s. Changes of injections; speeds are provided in Figure 3.

During all 117 saline OCT pullbacks, we observed two complications (1.7%): one complication (0.85%) was injection-related ventricular fibrillation, which was discontinued by electrical cardioversion, and the procedure was successfully completed; another (0.85%) was extension catheter-related artery dissection in the diseased segment, which was successfully stented. We did not register any transient ECG changes or episodes of chest pain.

## Discussion

Despite the numerous benefits of OCT, its utilization in patients with impaired kidney function is limited due to the heightened risk of contrast-induced nephropathy (CIN).^26–28^ The objective of our study was to demonstrate that flushing techniques with normal saline can produce high-quality images, thereby reducing the need for contrast during OCT-guided procedures and broadening the application of this imaging modality. Our findings revealed that normal saline injections using an automatic pump at controlled volume and speed can yield adequate-quality images. Due to differences in viscosity between normal saline and contrast media (CM), normal saline must be injected at a higher speed and in a greater total volume. We provide recommendations for normal saline injection volumes and speeds that have not been previously published.

Optical coherence tomography provides visualization of the coronary artery microstructure at a resolution 10 times better than IVUS, allowing for microscopic assessment of artery structures such as edge dissections, tissue coverage of stent struts, and malposition, which cannot be adequately assessed by IVUS.^29^ Consequently, OCT has become the second most commonly used intracoronary imaging technique, with increasing utilization over the years. However, despite its advantages, OCT imaging requires flush media for blood clearance to achieve visualization with near-infrared light, typically utilizing contrast media (CM).^29–32^ This reliance on contrast media poses a significant limitation to OCT usage, particularly for patients with underlying CKD and a higher risk of contrast-induced nephropathy (CIN).^25,29,32^

Comparing different studies, a trend of decreasing contrast media usage for OCT procedures over time can be observed. For instance, in the iSIGHT study, the total contrast volume used in the OCT group was significantly higher than in the angiography group (94.10 ± 40.54 mL [27.63 ± 7.39 mL due to OCT] vs 72.3 ± 35.8 mL, *p* = .018), but not significantly different from the contrast volume used in the IVUS group (82.1 ± 41.3 mL, *p* = .277). An average of 12.85 ± 3.05 mL of contrast was used per OCT run, with no significant differences observed between pre-PCI and post-PCI runs (12.44 ± 2.81 vs 13.0 ± 3.11 mL, *p* = .218). Furthermore, OCT runs in the left coronary system required more contrast than imaging of the right coronary artery (14.45 ± 2.52 vs 10.62 ± 2.21 mL, *p* < .001).^33^ In comparison with other studies, the iSIGHT study demonstrated a notably smaller average contrast volume of 94 mL for OCT-guided PCI, significantly less than that used in the ILUMIEN III (222 mL)^34^ and OPINION (164 mL)^35^ studies. Moreover, the mean contrast volume (94 mL) used in the iSIGHT study for OCT-guided PCI cases was nearly two times smaller than that used in the angiography-guided PCI of ILUMIEN III (183 mL)^34^ and much smaller than that used in the IVUS arm of OPINION (138 mL) ,^35^ even considering the higher complexity of lesions treated in the iSIGHT study.^33^

In an effort to reduce the volume of contrast media (CM) used for OCT-guided procedures, several alternative flushing agents have been investigated in vivo.^36^ Low-molecular-weight dextran, with a viscosity very similar to contrast, has been considered as a potential flushing agent for achieving high-quality imaging.^37,38^ However, dextran poses a risk of renal injury as it is excreted from the kidneys unmetabolized, similar to contrast.^38,39^ Carbon dioxide, previously utilized for peripheral angiography,^40^ is not employed for intracoronary imaging due to concerns about inducing myocardial ischemia. Clear crystalloid solutions, such as Ringer’s solution, have been used in older occlusive OCT pullback techniques, demonstrating effective imaging quality.^20,41^ More recently, three small studies have investigated normal saline as a suitable flushing medium for OCT imaging.^42–44^ However, these studies utilized manual saline injections for imaging, lacking a clear protocol for achieving high-quality images. Nevertheless, no significant complications were documented with this imaging technique. Nalin et al. conducted a study with 27 patients undergoing intracoronary OCT imaging using manual injections of normal saline, but only 61% of the images were deemed suitable for analysis.^42^ Two other studies compared vessel measurements between OCT imaging with contrast media and normal saline, finding no significant differences in vessel diameter measurements between the two techniques.^43,44^

Our study demonstrated that normal saline (NS) might serve as an effective, non-nephrotoxic alternative as a flushing material for OCT imaging. However, the lower rate of high-quality Oi images after PCI may be attributed to the increased lumen area and imaging lesion length post-procedure. This challenge was easily overcome by adjusting the injection speed in post-PCI imaging pullbacks. For OCT imaging with contrast media (CM), the contrast flush rate is typically set at 4 mL/s for a total volume of 14–16 mL for the left coronary artery (LCA) and 3 mL/s for a total volume of 12–14 mL for the right coronary artery (RCA).^25^ NS, having a lower viscosity compared to contrast, allows for higher injection speed limits for flushing injections. The NS flushing injection speed depends on the length of the imaging segment and the size of the vessel; however, even repeated runs did not have any significant impact on the patient.

Repeated injections of NS may have a negative cumulative effect on patients with reduced left ventricle ejection fraction (LVEF) and/or heart failure (HF) symptoms. The maximal flushing speed of 7 mL/s was used only once in our study, with 6 mL/s deemed a safe and effective speed limit. During standard OCT imaging involving two pullbacks (pre and post PCI), an average of up to 40 mL may be injected. However, if multiple imaging pullback runs are repeated, the amount of NS can exceed 100 mL or more. This increase in fluid volume may have a negative effect on patients with reduced LVEF and HF symptoms. Although our study did not specifically analyze patients with LV EF as a subgroup, the potential negative impact on their HF status should be considered if multiple repeated pullbacks with NS are performed.

Complications related to OCT are rare (0.6%) and typically self-limiting. They include transient chest pain and QRS widening/ST-depression/elevation, ventricular fibrillation due to catheter occlusion and/or deep guide catheter intubation, air embolism, and vessel dissection.^36,45–47^ Ventricular fibrillation is the most common arrhythmia, with an incidence as high as 1.1%.^47^ A recent study by Terada et al. revealed that contrast volume was the only independent predictor of ventricular fibrillation, with a cut-off value of 19.2 mL for predicting arrhythmia (area under the curve, 0.713, *p* < .001; diagnostic accuracy, 87.1%).^48^ In our study, we demonstrated that our proposed protocol for normal saline injection during the OCT pullback is safe. We did not register any procedure-related major complications, even with high volume and saline injection rates.

Obtaining good-quality images often necessitates elective target vessel engagement with an extension guiding catheter or a 5 to 6 French “mother-in-child” catheter to achieve adequate clearance, particularly in lesions with a larger diameter. While our suggested protocol has shown effectiveness and safety, larger-scale randomized clinical trials are needed to confirm the findings of our study.

## Study limitations

The major limitation of this study is the absence of a control group, in which patients undergoing OCT would have received traditional flushing with contrast media (CM). This comparison would have allowed for demonstrating the potential benefits of the method, such as reduced CM volume used for the procedure and a possible decrease in the development of contrast-induced nephropathy (CIN). The lack of a control group diminishes the efficacy of assessing the safety of the saline OCT method. However, we compared the complication rate of OCT with saline to that of traditional OCT methods using CM, as reported in published clinical trials. For further evaluation of the safety and efficacy of our proposed method, additional randomized studies with a larger sample size are necessary.

## Conclusions

This study demonstrated that heparinized saline injections for OCT imaging are effective in generating good-quality OCT images suitable for clinical use and assessment. Heparinized saline flushing injections at rates of 4 and 7 mL/s were found to be safe and well tolerated by patients, with no significant complications observed. However, the results of this pilot study need to be confirmed in multicenter randomized controlled trials.

## References

[1] A Manuscript and C Incidence . Tsai 14 (AKI from PCI 288,000). 7(2009): 1–9, 2014.

[2] Rihal CS Textor SC Grill DE , et al. Incidence and prognostic importance of acute renal failure after percutaneous coronary intervention. Circulation 2002; 105(19): 2259–2264. 12010907 10.1161/01.cir.0000016043.87291.33

[3] Parikh PB Jeremias A Naidu SS , et al. Impact of severity of renal dysfunction on determinants of in-hospital mortality among patients undergoing percutaneous coronary intervention. Catheter Cardiovasc Interv 2012; 80(3): 352–357. 22566286 10.1002/ccd.23394

[4] Gruberg L Mintz GS Mehran R , et al. The prognostic implications of further renal function deterioration within 48 h of interventional coronary procedures in patients with pre-existent chronic renal insufficiency. J Am Coll Cardiol 2000; 36(5): 1542–1548. 11079656 10.1016/s0735-1097(00)00917-7

[5] Gruberg L Mehran R Dangas G , et al. Acute renal failure requiring dialysis after percutaneous coronary interventions. Catheter Cardiovasc Interv 2001; 52(4): 409–416. 11285590 10.1002/ccd.1093

[6] Chertow GM Burdick E Honour M , et al. Acute kidney injury, mortality, length of stay, and costs in hospitalized patients. J Am Soc Nephrol 2005; 16(11): 3365–3370. 16177006 10.1681/ASN.2004090740

[7] Laskey WK Jenkins C Selzer F , et al. Volume-to-creatinine clearance ratio. a pharmacokinetically based risk factor for prediction of early creatinine increase after percutaneous coronary intervention. J Am Coll Cardiol 2007; 50(7): 584–590. 17692741 10.1016/j.jacc.2007.03.058

[8] Gurm HS Dixon SR Smith DE , et al. Renal function-based contrast dosing to define safe limits of radiographic contrast media in patients undergoing percutaneous coronary interventions. J Am Coll Cardiol 2011; 58(9): 907–914. 21851878 10.1016/j.jacc.2011.05.023

[9] Iannaccone M D'Ascenzo F Frangieh AH , et al. Impact of an optical coherence tomography guided approach in acute coronary syndromes: a propensity matched analysis from the international FORMIDABLE-CARDIOGROUP IV and USZ registry. Catheter Cardiovasc Interv 2017; 90(2): E46–E52. 10.1002/ccd.26880 28029210

[10] Meneveau N Souteyrand G Motreff P , et al. Optical coherence tomography to optimize results of percutaneous coronary intervention in patients with non-ST-elevation acute coronary syndrome: results of the multicenter, randomized DOCTORS study (Does Optical Coherence Tomography Optimize Results of Stenting). Circulation 2016; 134(13): 906–917. 27573032 10.1161/CIRCULATIONAHA.116.024393

[11] Claessen BE Mehran R Mintz GS , et al. Impact of intravascular ultrasound imaging on early and late clinical outcomes following percutaneous coronary intervention with drug-eluting stents. JACC Cardiovasc Interv 2011; 4(9): 974–981. 21939937 10.1016/j.jcin.2011.07.005

[12] Kim BK Shin DH Hong MK , et al. Clinical impact of intravascular ultrasound-guided chronic total occlusion intervention with zotarolimus-eluting versus biolimus-eluting stent implantation randomized study. Circ Cardiovasc Interv 2015; 8(7): e002592. 26156151 10.1161/CIRCINTERVENTIONS.115.002592

[13] Choi KH Song YB Lee JM , et al. Impact of intravascular ultrasound-guided percutaneous coronary intervention on long-term clinical outcomes in patients undergoing complex procedures. JACC Cardiovasc Interv 2019; 12(7): 607–620. 30878474 10.1016/j.jcin.2019.01.227

[14] Wakabayashi K Lindsay J Laynez-Carnicero A , et al. Utility of intravascular ultrasound guidance in patients undergoing percutaneous coronary intervention for type c lesions. J Interv Cardiol 2012; 25(5): 452–459. 22672141 10.1111/j.1540-8183.2012.00744.x

[15] Kim JS Hong MK Ko YG , et al. Impact of intravascular ultrasound guidance on long-term clinical outcomes in patients treated with drug-eluting stent for bifurcation lesions: data from a Korean multicenter bifurcation registry. Am Heart J 2011; 161(1): 180–187. 21167352 10.1016/j.ahj.2010.10.002

[16] Prati F Di Vito L Biondi-Zoccai G , et al. Angiography alone versus angiography plus optical coherence tomography to guide decision-making during percutaneous coronary intervention: the Centro per la Lotta contro l’Infarto-Optimisation of Percutaneous Coronary Intervention (CLI-OPCI) study. EuroIntervention 2012; 8(7): 823–829. 23034247 10.4244/EIJV8I7A125

[17] Jones DA Rathod KS Koganti S , et al. Angiography alone versus angiography plus optical coherence tomography to guide percutaneous coronary intervention: outcomes from the Pan-London PCI cohort. JACC Cardiovasc Interv 2018; 11(14): 1313–1321. 30025725 10.1016/j.jcin.2018.01.274

[18] Di Vito L Yoon JH Kato K , et al. Comprehensive overview of definitions for optical coherence tomography-based plaque and stent analyses. Coron Artery Dis 2014; 25(2): 172–185. 24356250 10.1097/MCA.0000000000000072

[19] Prati F Guagliumi G Mintz GS , et al. Expert review document part 2: methodology, terminology and clinical applications of optical coherence tomography for the assessment of interventional procedures. Eur Heart J; 33: 2513. 10.1093/eurheartj/ehs095 PMC3470836 22653335

[20] Prati F Regar E Mintz GS , et al. Expert review document on methodology, terminology, and clinical applications of optical coherence tomography: physical principles, methodology of image acquisition, and clinical application for assessment of coronary arteries and atherosclerosis. Eur Heart J 2010; 31(4): 401–415. 19892716 10.1093/eurheartj/ehp433

[21] Yamaguchi T Terashima M Akasaka T , et al. Safety and feasibility of an intravascular optical coherence tomography image wire system in the clinical setting. Am J Cardiol 2008; 101(5): 562–567. 18307999 10.1016/j.amjcard.2007.09.116

[22] Choma M Sarunic M Yang C , et al. Sensitivity advantage of swept source and Fourier domain optical coherence tomography. Opt Express 2003; 11(18): 2183–2189. 19466106 10.1364/oe.11.002183

[23] Kataiwa H Tanaka A Kitabata H , et al. Safety and usefulness of non-occlusion image acquisition technique for optical coherence tomography. Circ J 2008; 72(9): 1536–1537. 18724035 10.1253/circj.cj-08-0406

[24] Roleder T Jąkała J Kałuża GL , et al. The basics of intravascular optical coherence tomography. Postep w Kardiol Interwencyjnej 2015; 11(2): 74–83. 10.5114/pwki.2015.52278 PMC4495121 26161097

[25] Ali ZA Karimi Galougahi K Mintz GS , et al. Intracoronary optical coherence tomography: state of the art and future directions. EuroIntervention 2021; 17(2): E105–E123. 34110288 10.4244/EIJ-D-21-00089 PMC9725016

[26] Au TH Bruckner A Mohiuddin SM , et al. The prevention of contrast-induced nephropathy. Ann Pharmacother 2014; 48(10): 1332–1342. DOI: 10.1177/1060028014541996. 24994723

[27] Morcos SK . Prevention of contrast media–induced nephrotoxicity after angiographic procedures. J Vasc Interv Radiol 2005; 16(1): 13–23. 15640403 10.1097/01.RVI.0000145224.02920.C2

[28] Vasu N Subban V Ajit Mullasari S . Zero contrast optical coherence tomography–guided percutaneous coronary intervention for in-stent restenosis of the saphenous vein graft using a non-contrast flush medium. Indian Heart J 2018; 70(Suppl 3): S492–S495. 30595315 10.1016/j.ihj.2018.11.001 PMC6309879

[29] Räber L Mintz GS Koskinas KC , et al. Clinical use of intracoronary imaging. Part 1: guidance and optimization of coronary interventions. An expert consensus document of the European Association of Percutaneous Cardiovascular Interventions. EuroIntervention 2018; 14(6): 656–677. 29939149 10.4244/EIJY18M06_01

[30] Machanahalli Balakrishna A Ismayl M Walters RW , et al. Comparing optical coherence tomography and intravascular ultrasound guidance for percutaneous coronary intervention: trends and outcomes 2010-2019. Curr Probl Cardiol 2022; 47(9): 101270. 35640848 10.1016/j.cpcardiol.2022.101270

[31] Huang D Swanson EA Lin CP , et al. Optical coherence tomography. Science 1991; 254(5035): 1178–1181. 1957169 10.1126/science.1957169 PMC4638169

[32] Araki M Park SJ Dauerman HL , et al. Optical coherence tomography in coronary atherosclerosis assessment and intervention. Nat Rev Cardiol 2022; 19(10): 684–703. 35449407 10.1038/s41569-022-00687-9 PMC9982688

[33] Chamié D Costa JR Jr Damiani LP , et al. Optical coherence tomography versus intravascular ultrasound and angiography to guide percutaneous coronary interventions: the iSIGHT randomized trial. Circ Cardiovasc Interv 2021; 14(3): E009452. 33685212 10.1161/CIRCINTERVENTIONS.120.009452

[34] Ali ZA Maehara A Généreux P , et al. Optical coherence tomography compared with intravascular ultrasound and with angiography to guide coronary stent implantation (ILUMIEN III: OPTIMIZE PCI): a randomised controlled trial. Lancet 2016; 388(10060): 2618–2628. 27806900 10.1016/S0140-6736(16)31922-5

[35] Kubo T Shinke T Okamura T , et al. Optical frequency domain imaging vs. intravascular ultrasound in percutaneous coronary intervention (OPINION trial): one-year angiographic and clinical results. Eur Heart J 2017; 38(42): 3139–3147. 29121226 10.1093/eurheartj/ehx351 PMC5837511

[36] Suter MJ Kashiwagi M Gallagher KA , et al. Optimizing flushing parameters in intracoronary optical coherence tomography: an in vivo swine study. Int J Cardiovasc Imaging 2015; 31(6): 1097–1106. 25922149 10.1007/s10554-015-0668-0 PMC4490049

[37] Ozaki Y Kitabata H Tsujioka H , et al. Comparison of contrast media and low-molecular-weight dextran for frequency-domain optical coherence tomography. Circ J 2012; 76(4): 922–927. 22301848 10.1253/circj.cj-11-1122

[38] Vijayvergiya R Ratheesh KJ Gupta A . Low molecular weight Dextran: an alternative to radiographic contrast agent for optical coherence tomography imaging. IHJ Cardiovasc. Case Reports 2017; 1(1): 10–11.

[39] Mailloux L Swartz CD Capizzi R , et al. Acute renal failure after administration of low-molecular-weight dextran. N Engl J Med 1967; 277(21): 1113–1118. 6054998 10.1056/NEJM196711232772103

[40] Hawkins IF . Carbon dioxide digital subtraction arteriography. AJR Am J Roentgenol 1982; 139(1): 19–24. 6807073 10.2214/ajr.139.1.19

[41] Kawase Y Hoshino K Yoneyama R , et al. In vivo volumetric analysis of coronary stent using optical coherence tomography with a novel balloon occlusion-flushing catheter: a comparison with intravascular ultrasound. Ultrasound Med Biol 2005; 31(10): 1343–1349. 16223637 10.1016/j.ultrasmedbio.2005.05.010

[42] Mahesh NK Gupta A Barward P , et al. Study of saline optical coherence tomography-guided percutaneous coronary intervention (SOCT-PCI Study). Indian Heart J 2020; 72(4): 239–243. 32861376 10.1016/j.ihj.2020.03.013 PMC7474129

[43] Gore AK Shlofmitz E Karimi Galougahi K , et al. Prospective comparison between saline and radiocontrast for intracoronary imaging with optical coherence tomography. JACC Cardiovasc Imaging 2020; 13(9): 2060–2062. 32563662 10.1016/j.jcmg.2020.04.018

[44] Gupta A Chhikara S Vijayvergiya R , et al. Saline as an Alternative to radio-contrast for optical coherence tomography-guided percutaneous coronary intervention: a prospective comparison. Cardiovasc Revasc Med 2022; 34: 86–91. 33468422 10.1016/j.carrev.2021.01.010

[45] Kubo T Akasaka T Shite J , et al. OCT compared with IVUS in a coronary lesion assessment: the OPUS-CLASS study. JACC Cardiovasc Imaging 2013; 6(10): 1095–1104. 24011777 10.1016/j.jcmg.2013.04.014

[46] Van Der Sijde JN Karanasos A van Ditzhuijzen NS , et al. Safety of optical coherence tomography in daily practice: a comparison with intravascular ultrasound. Eur Heart J Cardiovasc Imaging 2017; 18(4): 467–474. 26992420 10.1093/ehjci/jew037

[47] Barlis P Gonzalo N Di Mario C , et al. A multicentre evaluation of the safety of intracoronary optical coherence tomography. EuroIntervention 2009; 5(1): 90–95. 19577988 10.4244/eijv5i1a14

[48] Terada N Kuramochi T Sugiyama T , et al. Ventricular fibrillation during optical coherence tomography/optical frequency domain imaging - a large single-center experience. Circ J 2020; 84(2): 178–185. 31941850 10.1253/circj.CJ-19-0736

